# Potential roles of aspartic acid D-isomerization in proteins implicated in the pathogenesis of Alzheimer’s disease

**DOI:** 10.4103/NRR.NRR-D-25-01311

**Published:** 2026-02-05

**Authors:** Genta Ito, Naoko Utsunomiya-Tate

**Affiliations:** Department of Biomolecular Chemistry, Faculty of Pharmaceutical Sciences, Teikyo University, Tokyo, Japan

**Figure 1:**
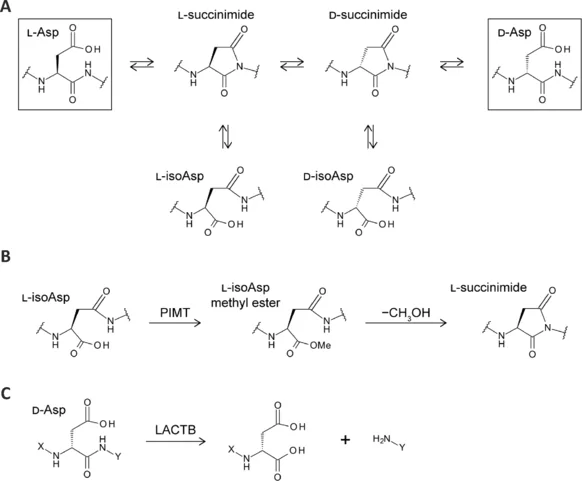
Non-enzymatic isomerization of aspartic acid in proteins, and proteins that protect against Asp isomerization. (A) In proteins, an L-Asp residue undergoes non-enzymatic isomerization to form L-isoAsp, D-Asp, and D-isoAsp. (B) PIMT methylates the carboxy group of the L-isoAsp residue, facilitating its conversion to a L-succinimide intermediate. (C) LACTB hydrolyzes the C-terminal peptide bond of the D-Asp residue. X and Y are neighboring residues of the D-Asp residue. Asp: Aspartic acid residue; D-Asp: D-aspartic acid; D-isoAsp: D-isoaspartate; LACTB: serine β-lactamase-like protein; L-Asp: L-aspartic acid; L-isoAsp: L-isoaspartate; PIMT: protein L-isoaspartyl methyltransferase.

**Non-enzymatic isomerization of aspartic acid residues in proteins associated with Alzheimer’s disease:** Proteins are composed of L-amino acids following ribosomal translation. However, if they remain unmetabolized for an extended period, some residues (particularly aspartic acid [Asp]) undergo isomerization to the D-form. This process begins with nucleophilic attack (by the nitrogen atom of the amide group of an adjacent residue) on the side-chain carboxy group of an Asp residue. This forms a five-membered L-succinimide intermediate (**Figure 1A**), which has two carbonyl carbons and can transform back to an L-Asp residue or isomerize into an L-isoAsp residue. The chiral center of the L-succinimide intermediate can easily invert to form a D-succinimide intermediate via keto-enol tautomerization. This D-succinimide intermediate then cleaves to yield a D-aspartic acid (D-Asp) or D-isoaspartate (D-isoAsp) residue (**Figure 1A**).

Asp isomerization is usually undetectable because the turnover time of proteins is typically only a few days, much faster than the time required for Asp isomerization. However, certain proteins with half-lives ranging from days to months undergo Asp isomerization. Consequently, isomerized Asp residues are frequently observed in proteins in the eye lens, tooth dentin, and neuronal myelin; they also accumulate with aging (Man et al., 1983). Asp isomerization in proteins can alter their three-dimensional structure, interactions with binding partners, and protein functions, potentially leading to age-related disorders. Isomerized Asp has been detected in crystallins, which accumulate in the lenses of patients with cataracts. It has also been found in amyloid-β (Aβ) peptides and tau proteins, which accumulate in the brains of patients with Alzheimer’s disease (AD). These proteins undergo misfolding and fibrillization due to unknown mechanisms with aging, forming accumulations of insoluble aggregates in disease lesions. It has been hypothesized that the aggregation process of these proteins is partly triggered by a conformational change caused by Asp isomerization.

Some proteins have been shown to exhibit protective properties against Asp isomerization in proteins; one example is protein L-isoaspartyl methyltransferase (Kim et al., 1997). Protein L-isoaspartyl methyltransferase transfers a methyl group to the carboxy group of an L-isoAsp residue to form a methyl ester (**Figure 1B**). This process converts the L-isoAsp residue into a five-membered L-succinimide intermediate, which then reverts to an L-Asp residue. As *Pimt* knockout mice die from fatal seizures a few months after birth, the accumulation of L-isoAsp-containing proteins may negatively impact the regulation of neuroexcitability. Several L-isoAsp-containing neuronal proteins have been identified in the brains of *Pimt* knockout mice, including synapsin-1, which localizes to presynaptic terminals. However, their role in the development of seizures remains to be determined.

Recently, we discovered that the human protein, serine β-lactamase-like protein LACTB, exhibits D-aspartyl endopeptidase (DAEP) activity *in vitro* (Ito and Utsunomiya-Tate, 2025). LACTB selectively hydrolyzes C-terminal peptide bonds to D-Asp. This enzymatic activity suggests a potential protective role for LACTB, acting to cleave proteins containing D-Asp and promote their degradation (**Figure 1C**). Notably, the genomic locus containing the *LACTB* gene has been associated with AD in a genome-wide association study (Jansen et al., 2019). In this perspective, we summarize the effects of Asp isomerization in Aβ and tau and discuss the potential role of LACTB in AD pathogenesis.

**Figure 2:**
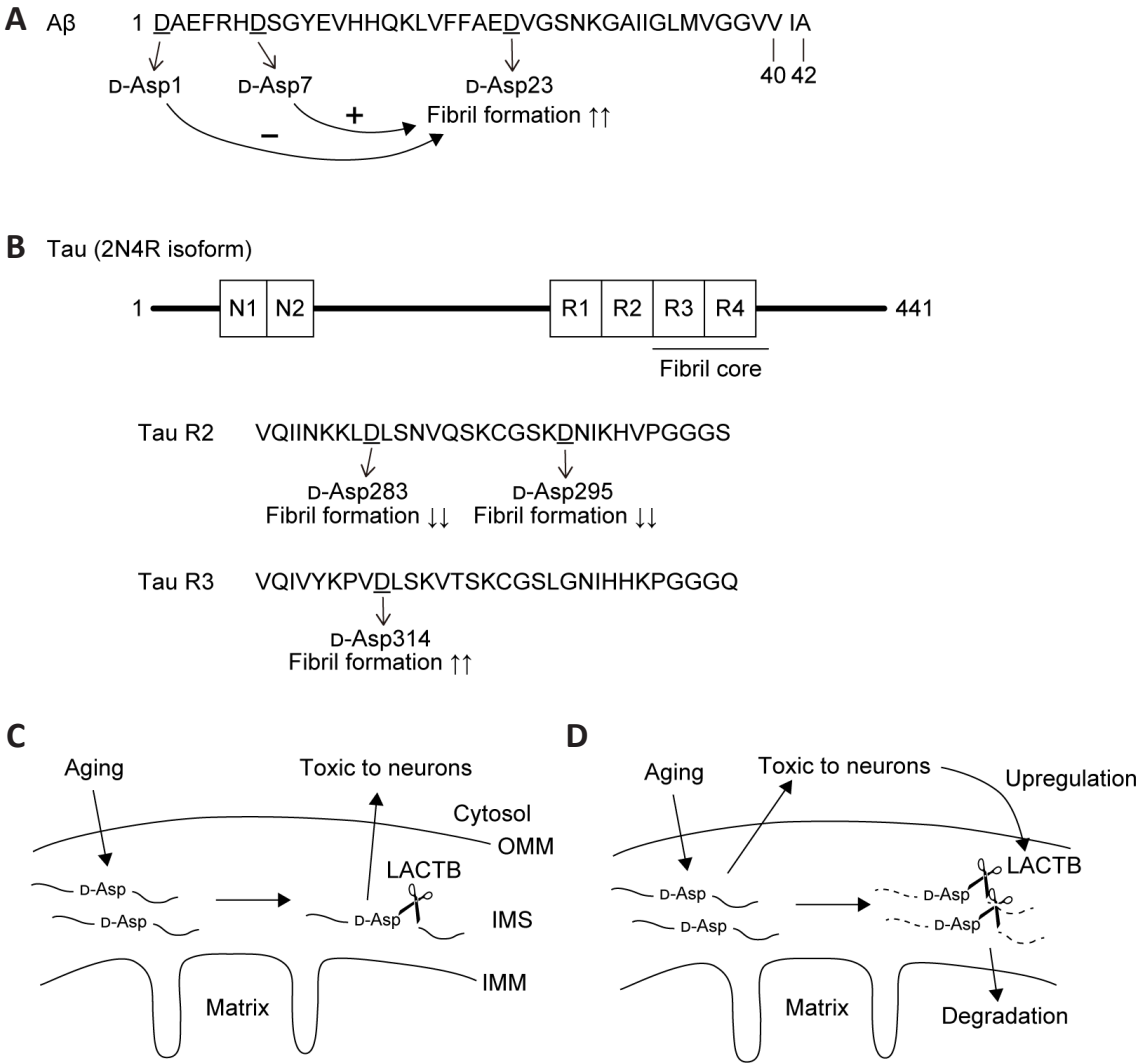
Effects of Asp D-isomerization in Aβ and tau, and hypothetical models of the role of LACTB in neurodegeneration. (A) The 40- and 42-amino acid sequences of Aβ and the effects of Asp D-isomerization on fibril formation. (B) Domain architecture of full-length tau protein (2N4R), which contains two N-terminal insertions (N1 and N2) and four repeat sequences (R1–R4). The sequences of the tau R2 and R3 regions, as well as the effects of Asp D-isomerization on fibril formation, are shown below. (C) Model 1: Cleavage of D-Asp-containing proteins by LACTB produces neurotoxic byproducts. (D) Model 2: D-Asp-containing proteins generated during aging are toxic to neurons. Expression of LACTB is increased to counteract the toxicity. Cleavage of these proteins by LACTB leads to their degradation, thereby protecting neurons. The scissors icons in C and D represent LACTB. Aβ: Amyloid-β; Asp: aspartic acid residue; D-Asp: D-aspartic acid; IMM: inner mitochondrial membrane; IMS: intermembrane space; LACTB: serine β-lactamase-like protein; OMM: outer mitochondrial membrane.

**Aspartic acid D-isomerization in amyloid-β and tau and its effect on fibril formation:** Abnormally accumulated Aβ peptides extracted from the brains of patients with AD exhibit a significant degree of isomerization to L-isoAsp and D-Asp. These levels range from 5% to 10% of total Aβ accumulated in AD brains (Shapira et al., 1988), which is considerably higher than the isomerization levels typically observed for normal proteins. The Aβ peptides that accumulate in AD brains are composed of 40 to 42 amino acids (**Figure 2A**). All these peptides contain three Asp residues: Asp1, Asp7, and Asp23. Among these residues, high proportions of isomerization at Asp7 to L-isoAsp and D-Asp have been reported (Roher et al., 1993). As previously demonstrated, Asp isomerization in Aβ exerts differential effects on β-sheet transition and fibril formation. D-isomerization of Asp23 (D-Asp23) can significantly accelerate Aβ fibril formation (Sakai-Kato et al., 2007; Sugiki and Utsunomiya-Tate, 2013), while this enhancement can be blocked by simultaneous D-isomerization of Asp1 (D-Asp1) or augmented by simultaneous D-isomerization of Asp7 (D-Asp7). Consequently, it is plausible that D-Asp7 and D-Asp23 present in Aβ trigger fibril formation in AD brains.

Asp D-isomerization has also been detected in tau proteins that accumulate as neurofibrillary tangles in the brains of patients with AD (Fisher et al., 1991). Tau is a larger cytosolic protein than Aβ, consisting of 352 to 441 amino acids, depending on the isoform produced by alternative splicing (**Figure 2B**). Tau has an intrinsically disordered domain containing three to four repeats of a 31 to 32 amino acid sequence (R1–R4 in **Figure 2B**). Full-length tau protein, as well as the repeat domain alone, has been shown to form amyloid fibrils *in vitro*, with the repeat domain constituting the core region of fibrils. This suggests that Asp isomerization in the repeat domain might affect tau fibril formation. When examined in isolated peptides, the second (R2) and third (R3) repeat sequences can form amyloid fibrils *in vitro*. Based on analyses using these peptides, D-isomerization of Asp283 (D-Asp283) and Asp295 (D-Asp295) in the R2 repeat sequence inhibits R2 fibril formation, whereas D-isomerization of Asp314 (D-Asp314) in the R3 repeat sequence promotes R3 fibril formation. To address the effects of Asp isomerization on fibril formation of full-length tau, it is necessary to use synthetic full-length tau proteins, or at least their repeat domain, harboring isomerized Asp. However, synthesizing such long polypeptides remains technically challenging.

The process by which intrinsically soluble Aβ and tau form insoluble fibrils and aggregates in AD brains remains unclear. Additionally, the temporal sequence of Asp isomerization in proteins remains to be elucidated. Specifically, it is not yet known whether Asp isomerization occurs before or after the accumulation of these proteins in the brain. One compelling hypothesis suggests that delayed metabolism and clearance of soluble Aβ and tau due to aging causes their accumulation harboring isomerized Asp, thereby triggering subsequent fibril formation. Collectively, D-isomerization of Asp has differential effects on fibril formation of Aβ and tau, depending on which Asp residue is isomerized. Future research should address whether D-isomerization of Asp occurs in soluble Aβ or tau proteins in the aging brain, triggering fibril formation.

**Potential involvement of LACTB, a D-aspartyl endopeptidase, in the pathogenesis of Alzheimer’s disease:** Mammalian DAEP activity has been identified in rabbit tissue homogenates (Kinouchi et al., 2004). However, the proteins responsible for this activity have not yet been identified. Our objective was to determine the molecular nature of mammalian DAEP activity. In this regard, we first predicted the three-dimensional structure of paenidase, the only known DAEP of bacterial origin, using AlphaFold2, an artificial intelligence-based algorithm for protein structure prediction (Ito and Utsunomiya-Tate, 2025). We then conducted a search of the AlphaFold2 structure database, which encompasses the human proteome, using the Dali server. We found that human LACTB has a structure that is strikingly similar to that of paenidase. Both paenidase and LACTB adopt a β-lactamase-like fold, which is conserved among many bacterial hydrolytic enzymes, including β-lactamase and D-Ala-D-Ala carboxypeptidase (Kelly and Kuzin, 1995). The three-dimensional structure of LACTB has been determined experimentally by cryogenic electron microscopy. The AlphaFold2 structure of paenidase and the cryogenic electron microscopy structure of LACTB were also well matched. We then expressed and purified recombinant LACTB protein from E. coli and examined DAEP activity using a generic fluorescent substrate, and a peptide substrate corresponding to Aβ_1–10_ containing D-Asp7 to measure paenidase DAEP activity. The LACTB protein demonstrated reasonably potent DAEP activity for both substrates. Additionally, LACTB DAEP activity was inhibited by an irreversible DAEP inhibitor (Kinouchi et al., 2004). A previous study has shown that mammalian DAEP is enriched in mitochondrial fractions, and LACTB has been identified as a mitochondrial protein (Polianskyte et al., 2009). Based on these observations, we concluded that LACTB is one of the proteins responsible for mammalian DAEP activity. LACTB may function as a defense mechanism against Asp D-isomerization in mitochondrial proteins.

The physiological function of LACTB is currently unknown. However, emerging evidence suggests that it plays an important role in the pathogenesis of AD and other age-related diseases, including cancer and metabolic disorders. Wingo et al. (2021) integrated the results of AD genome-wide association study with human brain proteomics, focusing on the dorsolateral prefrontal cortex to identify proteins associated with AD risk. They found that elevated levels of LACTB expression are linked to an increased risk of developing AD. These results imply that LACTB plays a significant role in AD pathogenesis. However, if LACTB plays a protective role against Asp D-isomerization in proteins, it is counterintuitive that an increase in LACTB expression causes AD. Additionally, as LACTB localizes to the mitochondrial intermembrane space, it is unlikely that it cleaves Aβ or tau containing D-Asp. Instead, LACTB may cleave proteins containing D-Asp present in the mitochondrial intermembrane space. Thus, the mechanism by which increased LACTB expression causes AD remains unclear. To explain this apparent discrepancy, two competing hypotheses are proposed (**Figure 2C** and **D**). One possibility is that a LACTB cleavage product plays a role in the neurodegenerative process of AD (**Figure 2C**). Alternatively, an increase in LACTB expression levels may be a compensatory reaction to overcome the neurodegenerative process triggered by D-Asp-containing proteins (**Figure 2D**). To determine the mechanism by which LACTB causes AD, it is necessary to identify the proteins cleaved by LACTB in the mitochondrial intermembrane space under pathologically relevant conditions.

**Conclusions:** Aβ and tau have been identified as causative proteins for AD, and therapeutics that target these pathological molecules are becoming available in clinics. However, the process by which they become toxic to neurons is still unclear. D-isomerization of amino acids in proteins is frequently observed in age-related disorders. However, the pathological roles of amino acid D-isomerization have not been thoroughly studied. Identifying the mechanisms that maintain the homochirality of amino acids within proteins is essential to understanding how their disruption leads to diseases such as AD. Nevertheless, the identification of LACTB as the first DAEP in human proteins brings us closer to understanding the role of Asp D-isomerization in proteins involved in the pathogenesis of AD.

*This work was supported by Advanced Comprehensive Research Organization (ACRO) Research Grant of Teikyo University (to GI and NUT).*

*This work is dedicated to the memory of Professor Naoko Utsunomiya-Tate, who passed away during the preparation of this manuscript.*
